# Can inverse probability treatment weighting (IPTW) be used to assess differences of CRBSI rates between non-tunneled femoral and jugular CVCs in PICU patients?

**DOI:** 10.1186/s12879-022-07571-4

**Published:** 2022-07-07

**Authors:** Khouloud Abdulrhman Al-Sofyani, Mohammed Shahab Uddin

**Affiliations:** 1 Department of Pediatric, Pediatric Intensive Care Unit, King Abdulaziz University Hospital, Faculty of Medicine and Clinical Skills and Simulation Center, King Abdulaziz University, Jeddah, Saudi Arabia; 2 Pediatric Department, Ministry of National Guard Health Affairs, Dammam, Saudi Arabia

**Keywords:** Catheter related blood stream infection (CRBSI), Central lines, Causal inference, Inverse probability treatment weighting, Propensity score, Children, Pediatric ICU

## Abstract

**Background:**

In children in the ICU, catheter-related bloodstream infections (CRBSI) have also been linked to mortality, morbidity, and healthcare costs. Although CRBSI poses many potential risks, including the need to avoid femoral access, there is debate regarding whether jugular access is preferable to femoral access in adults. Study reports support both perspectives. There is no consensus in meta-analyses. Children have yet to be examined in depth. Based on compliance with the central line bundle check lists, we aim to determine CRBSI risk in pediatric intensive care units for patients with non-tunneled femoral and internal jugular venous access.

**Methods:**

A retrospective cohort study was conducted on patients with central venous catheters in the pediatric ICU of King Abdulaziz University Hospital between January 1st, 2017 and January 30th, 2018. For the post-match balance, we use a standardized mean difference of less than 0.1 after inverse probability treatment weighting for all baseline covariates, and then we draw causal conclusions. As a final step, the Rosenbaum sensitivity test was applied to see if any bias influenced the results.

**Results:**

We recorded 145 central lines and 1463 central line days with 49 femoral accesses (33.79%) and 96 internal jugular accesses (66.21%). CRBSI per 1000 central line days are 4.10, along with standardized infections of 3.16. CRBSI risk differed between non-tunneled femoral vein access and internal jugular vein access by 0.074 (− 0.021, 0.167), P-value 0.06, and relative risk was 4.67 (0.87–25.05). Using our model, the actual probability was 4.14% (0.01–0.074) and the counterfactual probability was 2.79% (− 0.006, 0.062). An unobserved confounding factor was not identified in the sensitivity analysis.

**Conclusions:**

So long as the central line bundle is maintained, a femoral line does not increase the risk of CRBSI. Causation can be determined through propensity score weighting, as this is a trustworthy method of estimating causality. There is no better way to gain further insight in this regard than through the use of randomized, double-blinded, multicenter studies.

## Background

An estimated 15 million central lines are used in intensive care units (ICUs) every year. As per literature review, 31,000 Americans die every year from bloodstream infections related to central lines. Unfortunately, we're dealing with a preventable tragedy that's among the leading causes of death around the world. As a prime example, let's look at the catheter-related bloodstream infection (CRBSI) that happened 15 years after Err Is Human. As a result, over 80% of intensive care infections have been reduced since 1985 [1, 2]. It involved an adaptive intervention to change staff behavior and improve safety culture and teamwork, as well as five prevention practices that had been proven to prevent CRBSI [3–5]. Studies suggested that central line-associated bloodstream infections were associated with many severe health problems like prolonged hospitalization [6–9], increased mortality [10, 11], increased financial [12–15] and patient dissatisfaction. Researchers found CRBSI to be the culprit behind the costliest infections [16]. Additionally, the infection led to an increased in hospital mortality by 2.76 times, along with an increased in attributable mortality by 16% [17, 18]. A theory stated that lower extremity adult catheters carry an increased risk of infection because of all the skin flora at the site of insertion. Because of these concerns, current guidelines said to stay away from femoral central venous catheters (CVCs). It's because some published data suggests there's a greater chance of infection in the femoral vein after CVC [19–21]. A new approach was adopted for the following period to cut healthcare-associated bloodstream infection risks. According to this new idea, the risk of CRBSI is not dependent on where the central line is placed but instead dependent on how it is maintained [22, 23]. After this measure was adopted, the CRBSI rate dropped significantly in many qualities improvement projects, dropping from 1.65 per 1000 catheter-day before to 0.65 per 1000 catheter-day after the projects ended (P = 0.039) [24]. Additionally, meta-analysis data showed a 70% drop-in infection rate with a 95% confidence interval of 0.10 to 0.88. Since the introduction [25] of central line bundles, there has not been evidence of an increased risk of infection in femoral lines compared to the internal jugular [26–30]. In one randomized controlled trial, there was no difference in infection risk between patients using jugular or femoral vein access [31]. All of these studies involved adults. We are the first to evaluate pediatric CRBSI causal risk link based on central line location with inverse probability treatment weighting and propensity score matching.

## Methods: study design, setting, and population

**Fig. 1:**
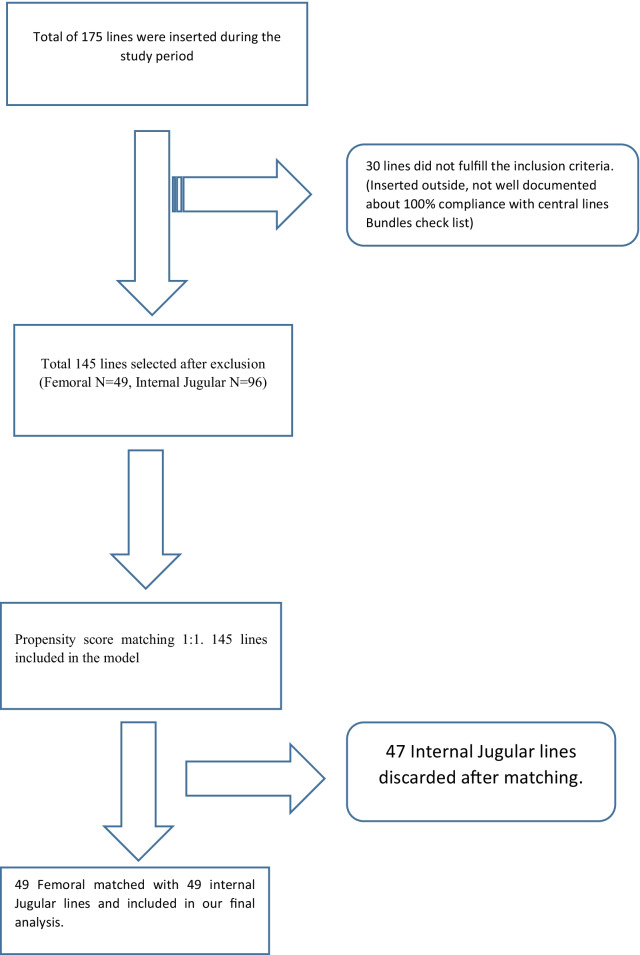
Flow chart of selection of the study cohort

Retrospective review of electronic medical records of pediatric ICU patients was conducted at King Abdulaziz University Hospital in Jeddah, Saudi Arabia. In our study, patients ranged in age from 1 month to 14 years old, and CVCs were inserted as an elective case, during an admission to the PICU from 1st January 2017 to 30th January 2018.Children in the pediatric intensive care unit, those with peripherally inserted central catheter (PICC), and central lines outside of the unit were excluded from the study. This project has been approved by the IRB (Institutional Review Board) under reference number HA-02-J-008. Figure 1 depicts the study population selection. Under the supervision of an experienced physician, each line was inserted using a CRBSI prevention bundle checklist and ultrasonography guidance. All lines exited at the mid neck or groin without tunneling. As per the intensive care unit central line bundle checklist, central lines were maintained in both locations. Only once a day, in the early morning, catheters were used for routine blood sampling. Antibiotic-impregnated catheters were not used. A goal-directed focus round was conducted, and timely decisions were made about the removal of the central line.

### Covariates

The following covariates were analyzed: age, gender, weight, total parenteral nutrition, and length of stay in a pediatric intensive care unit. Periods shorter than 10 days are coded as zeros, whereas longer periods are coded as ones. The number of days each line is exposed to the hospital environment was tracked with a variable that indicates time at risk for CRBSI. The variable is coded as zero if it's under 14 days. A code of 1 is assigned for a period longer than 14 days. It's either the femoral line or the internal jugular line. At the mid-neck, the jugular internal lines were exited, and the femoral internal lines were exited at the groin. These weren't tunneled lines. Our analysis ends with an event equal to zero if the CRBSI is negative, or an event equal to one if it's positive.

### CRBSI definition

Catheter-related bloodstream infection (CRBSI) is defined as the presence of bacteremia originating from an intravenous catheter [32].

### CRBSI diagnosis

The diagnosis of CRBSI was made by comparing colony counts in peripheral veins and catheter hubs with differential time to positivity (DTP) [33], a measurement of the time to positivity of blood cultures from peripheral veins and catheter hubs at the same time.

### Eligible central line (CL)

A central intravascular catheter tip is typically placed in the heart or within a major vein to infuse blood into, remove blood from, and monitor hemodynamics. For the purpose of investigating CRBSI events and counting central line device days in our study, internal jugular and femoral great vessels were taken into account. CRBSI events are considered eligible in pediatric intensive care if a central line remains in place longer than two consecutive calendar days or after the third day following the first entrance of the central line, or beyond the day of removal or discharge, whichever occurs first [34].

### Central lines days

During the period in which a central line is in place for a patient in the intensive care unit (ICU). A daily count of central lines, including all patients who have central lines, is done at 12 PM.

### Blood culture

Blood culture bottles BacT/ALERT SA Standard Aerobic (blue tap) and BacT/ALERT SN Standard Anaerobic (purple tap) were used in this study. Each bottle was inoculated with 5 ml of the patient’s blood sample collected from peripheral and a CVC lumen blood sample were withdrawn simultaneously from eligible lines. The culture bottles were further incubated in BacT/ALERT 3D (BioMérieux, Durham, NC, USA) till a positive signal was observed after a maximum incubation of 5 days. Gram staining was performed inside a safety cabinet Class II for positive blood culture bottles. Sabouraud dextrose agar (SDA) was used for yeast (Saudi Prepared Media Laboratories, Riyadh, KSA) along with Blood agar, Chocolate agar, and MacConkey agar for bacteria. Standard microbiological laboratory techniques and guide lines were followed for final culture reporting.

### Statistical analysis

All analysis were performed using R version 4.1.1 (2021-08-10), primarily the tableone package [35], to create "Table 1" for patient baseline characteristics. This package summarized both categorical and numerical variables. Categorical variables were counted or expressed as percentages. We used one of two methods to summarize continuous variables: a normally distributed method using means and standard deviations; or a non-normal method using medians and intervals. There are two standard hypothesis tests by default: the chi-square test for categorical variables and the one-way test for continuous variables. T-tests are equivalent to two-group ANOVAs. The exact argument specified Fisher tests for categorical variables, and Kruskal tests for nonnormal continuous variables. In the two-group case, Kruskal's test considered the equivalent of Wilcox's test. Standardized mean differences were measured with appropriate argument. We justified a P-value of less than 0.05 to be significant and a 95% confidence interval.

**Table 1:** Characteristics of unmatched and matched covariates with standardized mean difference (SMD) after propensity score weighting using inverse probability treatment weighting

Covariates	Unmatched			Matched		
	Internal jugular N = 96	Femoral N = 49	SMD	Internal Jugular N = 49	Femoral N = 49	SMD
Age [mean (SD)]	33.61 (47.48)	64.93 (57.63)	0.593	47.77 (59.22)	45.43 (51.87)	0.042
Gender (mean (SD))	0.41 (0.49)	0.47 (0.50)	0.127	0.43 (0.50)	0.42 (0.50)	0.023
Weight [mean (SD)]	10.01 (9.03)	16.82 (10.81)	0.684	13.34 (12.17)	12.87 (9.57)	0.043
TPN [mean (SD)]	0.11 (0.32)	0.18 (0.39)	0.193	0.18 (0.38)	0.17 (0.38)	0.014
CLdays [mean (SD)]	0.32 (0.42)	0.22 (0.42)	0.220	0.33 (0.47)	0.34 (0.48)	0.021
PICUStay [mean (SD)]	0.23 (0.42)	0.24 (0.43)	0.037	0.22 (0.42)	0.22 (0.42)	0.011

Standardized mean difference less than 0.1 is acceptable for excellent covariates balance for causal inference

In this study, we used the MatchIt package for matching propensity scores [36]. In order to balance baseline characteristics between femoral and internal jugular access groups, we calculated the propensity score. The model constructed using a multivariable logistic regression strategy, with the event as the dependent variable and all the baseline characteristics listed in Table 1 as covariates. The standardized mean differences [37] used to assess prematch imbalances and post-match balances for all baseline covariates. We considered a standardized mean difference of less than 0.1 excellent match. Weights calculated using propensity scores with the package ipw [38]. It balanced baseline characteristics between exposed and unexposed patients by weighing individuals according to their inverse probability of getting exposed to the treatment. For each individual, the weights calculated based on inverse probability treatment weighting as the exposed group was equal to 1/propensity score and the unexposed group was equal to 1/1 (1-propensity score). Hence, individuals who exposed but had a low probability of being exposed (as well as those who were not exposed but had a high probability of being exposed) were given larger weights, and their relative importance increased. The used of weights in the model allows the subsequent assignment of a sample to an exposed or unexposed group to be determined irrespective of the variables in the propensity score model. Pre and post matched cohorts with standardized mean differences are shown in Table 2. Lastly, outcome of interest analyzed by using the weighted data [39, 40] as causal relative risk and risk difference. Also, the stdReg package [41] utilized to analyze the attributable risk [42] of CRBSI for femoral lines.

**Table 2:** Final outcome analysis

Parameter	Estimate	Std error	95% CI	P-value
Causal relative risk	4.67	0.897	[0.872 to 25.05]	0.09
Causal risk difference	0.074	0.039	[− 0.019, 0.167]	0.06
Factual probability	0.0414	0.0166	[0.0088 to 0.0739]	X
Counterfactual probability	0.0279	0.0174	[− 0.0062, 0.0619]	X

Causal risk difference estimated from propensity score weighted data set using marginal structural model. Causal relative risk estimated from propensity score weights created from ps object generated by generalized boosted model and svyglm function extracted the output. Regression standardization stdReg R packages used to measure the factual, counterfactual probability

### Calculation of standardized infection ratio (SIR)

In order to track healthcare-associated infections (HAIs), the National Healthcare Safety Network used a summary measure called the Standardized Infection Ratio (SIR). It is calculated by dividing the observed infection rate by the predicted infection rate. Predicted infection rates were calculated by analyzing multivariable regression models derived from nationally aggregated data.

### Estimation of the causal diagram: we evaluated the causal effect of central line location on CRBSI using DAG analysis [43]

Age and weight were identified as two important adjustment sets that provided the most reliable estimates of the causal effect of central line location on CRBSI. The unobserved covariates had no effect on the causal pathway.

### Sensitivity test for estimation of unobserved covariates' impact

The rbounds package in R is used for the Rosenbaum Sensitivity Test [44] for Wilcoxon Signed Rank P-Value estimation to estimate hidden bias.

## Results

A total of 145 central lines and 1463 central line days were recorded. Median ages and weights of the study participants were 12 (4–72) months and 8.2 (4.4–17.5) kg, respectively. In this study, there were 83 male patients (57.2%), 62 female patients (42.8%) and 13.8% of patients got TPN. A total of 4.1 CRBSI cases were reported for every 1000 days on a central line. As per the National Healthcare Safety Network's negative binomial regression-based model, the number of CRBSI events for 1463 central line days was 1.90. Additionally, we observed an infection ratio of 3.16. Our sample had 96 internal jugular (66.21%) and 49 femoral (33.8%) lines. There were 111 (76.6%) patients in the PICU who stayed longer than 10 days. One hundred and three (71%) had central lines that lasted less than two weeks.

Following 1:1 propensity score matching and weighting, 98 central lines, 49 from each group, were analyzed (Fig. 1). Prior to applying propensity score weight, there was an imbalance between baseline covariates (Table 1). For example, the mean age of the treated group was 64.93 months, while the age of the control group was 33.61 months, indicating a high standardized mean difference (SMD) of 0.593. Moreover, the mean weight of the treated group was 16.82 compared to 10.01 for the control group. The standard mean difference was therefore 0.684, which was big. Nevertheless, TPN and gender were better balanced, with standardized mean differences of 0.127 and 0.189, respectively. Our goal was 0.1, so this is still unacceptable. PICU length of stay SMDs were 0.037 in both the femoral and internal jugular groups, indicating excellent balance. In both groups (Femoral and Internal Jugular Lines) the measured covariates were well balanced after weighting the propensity scores (Table 1). The histograms (Fig. 3) show the distribution of covariates and the love plots (Fig. 4) show the minimal standardized mean differences (less than 0.1) between the femoral and internal jugular groups before and after propensity score weighting of all variables. Based on propensity score weighting, it has shown excellent balance.

In a weighed generalized logistic model analysis, the estimated causal relative risk was 4.87, CI (0.872–25.05), P-value 0.09. Furthermore, the measured causal risk difference was 0.075, CI (− 0.019, 0.167), P-value 0.06 (Table 2). We calculated the number of bloodstream infections that could be avoided if no femoral lines were inserted during a child's stay in the pediatric intensive care unit. We used a standardized regression model that included age, weight, gender, TPN, PICU stay, and CL days to minimize bias and found that CLABSI has a factual probability of 4.14% (0.0088–0.0739) and a counterfactual probability of 2.79% (− 0.0061, 0.0619), assuming no femoral lines were inserted during the PICU stay (Table 2). We determined the hidden bias in our outcome analysis by using Rosenbaum's sensitivity test for Wilcoxon signed rank P-Value. A total of five gamma levels were used in the analysis. Using signed-rank P-values, we concluded that no unobserved confounding factors could affect the P-values.

## Discussion

The national guideline from 2002 says adults should not have a femoral central line because of the risk of CRBSI. But there is no scientific evidence to back that up. As a result of CDC/HICPAC and international guidelines [45, 46], the Institute for Healthcare Improvement recommended following "a central line bundle" for preventing catheter related blood stream infections [19, 46–49]. Subsequently, several studies examined whether or not site selection affected infectious risk while strictly following central line bundles. The recommendation encourages early removal as well as careful drapement and daily examination of all lines to ensure their necessity. In the years since central line bundles have been introduced, numerous studies have been conducted, but no correlation has been found between insertion site preference and infection risk [26, 28, 29]. In this study, we examined the research question: do catheter-related bloodstream infections differ much between non-tunneled jugular and femoral access? In what ways could inverse probability treatment weighting be used to draw conclusions from observational [50, 51] data? There have only been a few studies in the pediatric population that look at the same thing, as far as we know. We present a novel approach to investigating this question by comparing femoral and internal jugular lines to standard care, as well as compliance with the central line bundle, using inverse probability treatment weighting to balance covariates [18, 22, 52–55].

In fact, when estimating treatment effects from longitudinal observational studies, balancing weights are becoming more popular because people are not randomized [56–59]. A combination of probability score matching and inverse probability treatment weighting was used to balance the weights between groups (femoral versus internal jugular). Through balancing weights, we can create pseudo-randomized studies [60, 61] because we create comparable treatment groups based on key pretreatment characteristics that could have confounded treatment effect estimates. A causal diagram [43] was developed before we built a propensity score-based weighting model, so we could account for backdoor paths from covariates. Based on the causal diagram, site selection and bloodstream infection must be accounted for attributing causal impact to weight and age. On the other hand, TPN variables, PICU length of stay, central line days, how frequently a line was opened, and whether or not the patient was immunocompromised, have no effect on the causal pathway.

According to our study, the sample's median age was 12 months (IQR 4 to 72). It is lower than many other published data points [62–64], and men dominate, as they do in most research papers [64]. Furthermore, the CRBSI rate is noteworthy because it's higher than a few [62, 65] but lower than the majority of the research articles [63, 64, 66–68] In addition, we observed a CRBSI rate of 4.10 for 1463 central line days, but we predicted 1.902 for the same days. Therefore, our risk-adjusted standardized infection ratio was 3.155 [69, 70], compared with 1.3 for the National Healthcare Safety Network (NHSN) and the Centers for Disease Control and Prevention (CDC) [71]. The CDC defined 1 as the National Benchmark as of December 2010. In other words, a hospital with a CRBSI score of 1 does well since it means the score is the same as similar-sized or shaped hospitals [70–72]. Honestly, six CRBSI events in 13 months seems good to me. The CRBSI rate was not good enough when compared to a benchmark for the study period.

**Fig. 2:**
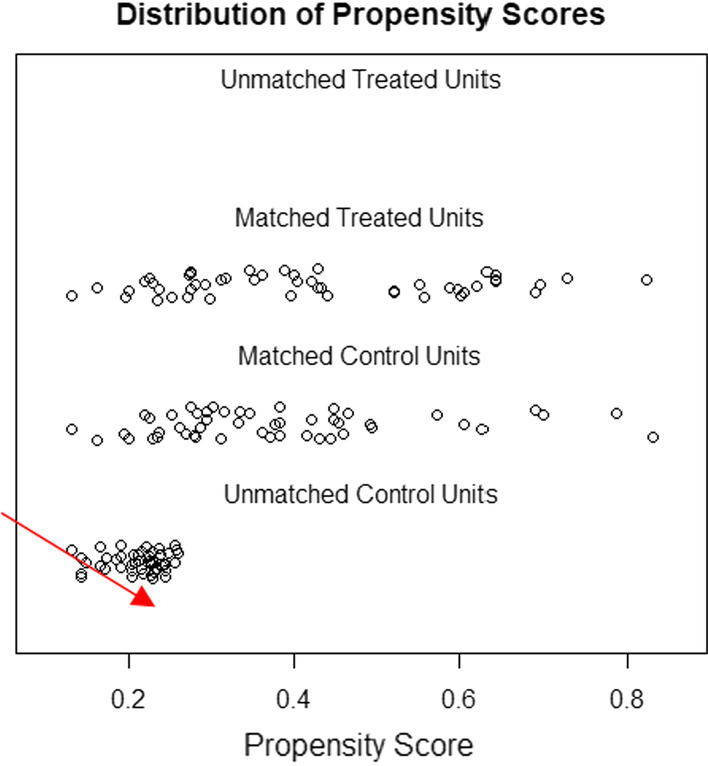
Distribution of propensity score after matching both treated (Femoral lines) and control (Internal jugular lines) after propensity score matching, 49 each group 1:1 match. Red arrow indicating unmatched internal jugular lines, 47 were discarded as no matching were found

**Fig. 3:**
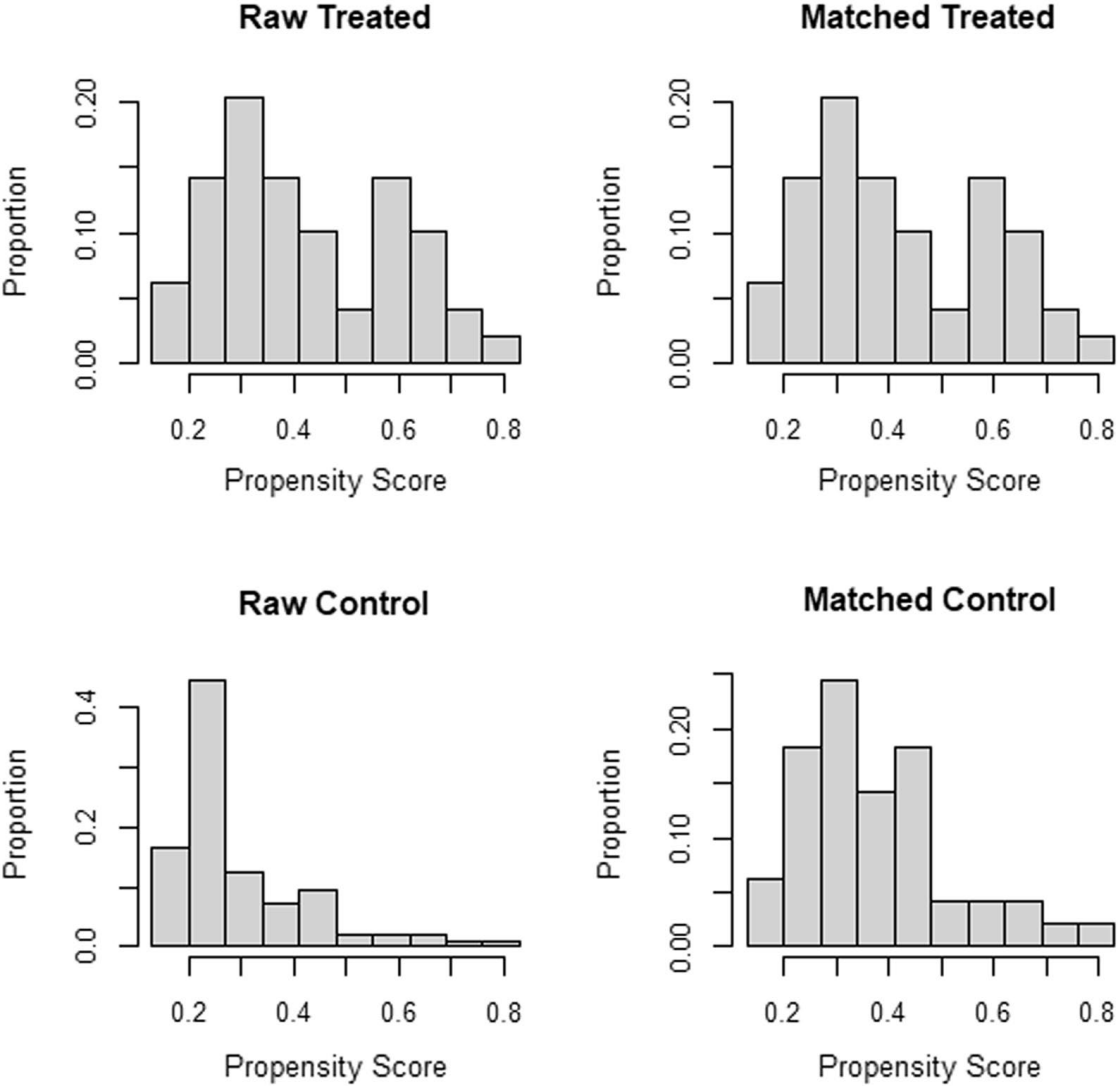
Histograms of propensity scores before and after matching

**Fig. 4:**
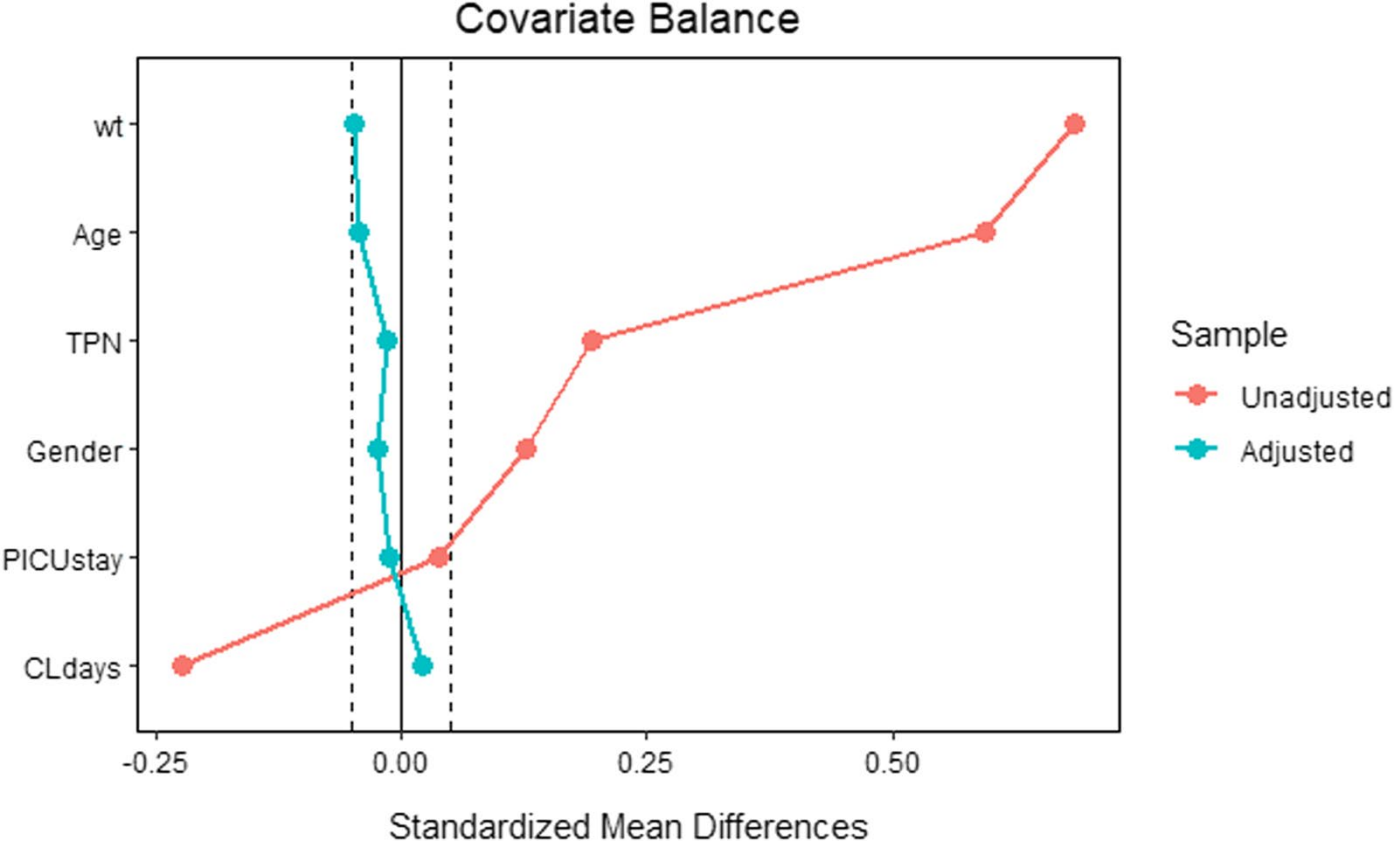
love-plot for demonstrating standardized mean difference between femoral lines and internal jugular lines groups before and after propensity score weighting for all variables

We included 96 internal jugular lines (control) and 49 femoral lines (treatment) with six covariates in the final matching model (Fig. 1). On the basis of the unmatched data, the mean age of the treated group was 64.93 months, and 33.61 months for the control group, resulting in a SMD of about 0.593. In fact, there was an imbalance in all covariates (SMD > 0.1), except for the length of stay in the PICU (SMD = 0.037) (Table 1). Interestingly, all covariates in both groups after post-matching ranged from 0.01 to 0.04 for standardized mean differences. (Table 1) Therefore, for casual inference estimation, this is a perfect balance for a 1:1 match between 49 femoral and 49 internal jugular lines. (Figs. 2, 3, 4).

The causal relative risk of CRBSI was estimated from the weighted data as 4.67, CI (0.872–25.05), P = 0.09 (Table 2). In other words, femoral lines are 4.67 times more likely to have CRBSI than internal jugular, but the difference is not statistically significant. Contrary to Goetz's (2016) published article with conflicting results, where she observed a CRBSI hazard ratio of 4.2; CI (2.0–8.8); P-value of 0.0001 [73]. There has been a similar finding in another author's work [74]. But Kumar (2009) reported catheter-related bloodstream infections were similar in femoral and internal jugular lines (2.3 vs 1.5 per 1000 catheter-days, respectively; P = 0.42). The jugular and femoral access sites are equally likely to cause catheter colonization, catheter-related bloodstream infection, and thrombosis in critically ill adult intensive care unit patients. This is contrary to the widely held belief that infections are more common at femoral access sites in adults. It does not make sense to extrapolate this to pediatric ICU patients because the recruited sample only includes adult patients on renal replacement therapy [31]. In addition, a systematic review showed that catheter-related bloodstream infections did not differ significantly between femoral and internal jugular sites. In this publication, the risk ratio was 1.35, with a 95% confidence interval of 0.84–2.19 and a P value of 0.2 [75]. Similarly, from December 2011 to June 2014, 3SITES, a randomized, controlled study involving four hospitals to the jugular group, the femoral group had an identical risk of CRBSI (hazard ratio, 1.3; 95% CI, 0.8 to 2.1; P = 0.31) [26]. While early studies suggested that femoral site access was associated with a higher risk of infection in adults, this has not been confirmed. As of this writing, research has shown no significant difference in the rate of CRBSI between these centers, assuming compliance with the central line bundles is maintained [76].

It is worth noting that the research discussed above focuses primarily on adult populations. There have been few articles in pediatrics that deal with this topic. The first study in King Abdulaziz Medical City, Riyadh, has been published. The researchers looked at catheter-related blood stream infection for catheters inserted in the PICU compared to operating rooms, and for catheters inserted in the femoral versus jugular or subclavian site (P*0.001) using a multiple logistic regression analysis [51]. Another interesting point is that 4512 CVC patients were enrolled in the second study. There was no evidence that any one of the sites had an increased risk of infection compared with the other sites, with hazard ratios of 0.951 (95% confidence interval [CI] 0.612–1.478) for the subclavian site, 0.956 (95% CI 0.593–1.541) for the internal jugular site, and 1.120 (95% CI 0.753–1.665) for the femoral site. Cox regression was used to adjust for age, severity of illness, and number of catheter days [50]. Recently, a prospective cohort study entitled "Central Venous Catheter Types and the Risk of Bloodstream Infection I n the Pediatric Intensive Care Unit: Two Years' Experience" was published in September 2021. A multiple logistic regression was used to capture the effects of preexisting compounding factors. Nevertheless, the results did not contradict our findings. OR 1.04, CI (0.49–3.49). Interestingly, the author included a central line bundle in the care process. In contrast to our study, Topal et al. (2021) had a larger sample size [77], but the median age and weight were similar to our patients' while infection rates were higher, at 6.2/1000 catheter days [78]. To learn more, Derderian et al. (2019) found that femoral lines had no significant risk factors for CRBSI in contrast to internal jugular lines, but they did show an association with venous thromboembolism [79]. Last but not least, Silvetti et al. (September 2017) enrolled an RCT on ClinicalTrials.gov that hypothesized that catheter colonization and CRBSI are less likely in the jugular insertion site than in the femoral insertion site in pediatric cardiac surgery [80]. The final results remain to be seen. We are looking forward to following this up in order to gain a deeper understanding of the experience.

Here, we looked at causality instead of associations. In addition, we have chosen to primarily focus on children as our sample population. To the best of our knowledge, an inverse probability treatment weighting based on propensity scores has never been reported previously for casually inferring CRBSI from non-tunneled femoral versus internal jugular vascular access. Furthermore, we used a standardized regression model to estimate the actual and counterfactual probability of CRBSI in femoral lines, which is another new approach. The factual probability of CRBSI was 4.14%, CI (0.09–7.49%), and the counterfactual probability was 2.79% (− 0.062%, 6.19%) (Table 2). The number of CRBSI during the observed period would not change if no femoral lines were inserted during the ICU stay or if the femoral line location was avoided. In combination with the standardized regression and the risk ratios from the weighted generalized multiple regression model, we found a risk difference of 0.074, CI (− 0.019, 0.169), P = 0.06, which supports our finding that there is no difference in CRBSI risk between non-tunneled femoral and internal jugular vascular access (Table 2). For instance, all the author used logistic regression to overcome the bias. Propensity-based weighting beats logistic regression on these fronts. It showed that the bias diminished as the number of events per confounder increased. In logistic regression, bias control depends entirely on the number of events and covariates. As an alternative, propensity score-based weighting could eliminate bias independent of events and covariates [81]. Finally, we believe another strength of our study is that our causality as a whole is not affected by unobserved covariates. We measured the Wilcoxon Signed Rank P-Value based on the Rosenbaum Sensitivity test [82], which estimates the hidden bias and explains the impact it has on our results. In spite of the high gamma levels, no unobserved confounding factors have been identified by the P-values in our study. Therefore, we were right to claim causality.

### Implications

There has been a gap in the research regarding CRBSI rates between femoral and internal jugular vascular access in pediatric intensive care. This study offers a new perspective for looking at this issue with an appropriate study design: a multicenter, double-blind, randomized control trial. Additionally, this is the first, a method used for estimating propensity score-based matching with inverse probability treatment weighting for causal inference, has been applied in a study. Furthermore, the article evaluates the attributable risk of bloodstream infections associated with the femoral central line, which is a unique approach in our article, and the beneficial effects of the central line bundle for preventing CRBSI, which have been further emphasized. In sum, our findings and the results of a few previous studies suggest that a central line associated with bloodstream infection placed in the femoral line is no more at risk than one placed in the jugular. Based on our results and findings of prior studies, we conclude that if the central line bundle is strictly maintained, there is no additional risk of bloodstream infection for the femoral line when compared to the internal jugular. It will be interesting to see how the enrolled RCT performs in this regard. We are very eager to pursue this opportunity to further our understanding and to have a rewarding experience.

*Some limitations* were encountered in this study. To begin with, we only collected data at one site, especially with few observations over short periods of time. Having a multicenter cohort is definitely superior to having a single-center, but our point estimate has a risk ratio similar to those that have been published in other studies. Second, since the study is an observational study, the causal inference does not have the same level of meaning as a randomized controlled trial (RCT), which is considered the gold standard in clinical research. Noteworthy, our treat and control groups balance metric SMD -values ranging 0.01to 0.04 after propensity score matching and weighting, which is only possible in a randomized clinical trial. Thus, we were correct in asserting causation. Moreover, the Rosenbaum Sensitivity Test for Wilcoxon Signed Rank P-Value for unobserved cofounding estimation and cause-and-effect diagrams indicate that our research provides strong evidence for true causality. The third point is that we cannot comment on several potentially important infection control practices that applied to both the femoral and internal jugular, including the length of the lines, the frequency with which each central line was open, and the same-level skilled provider inserting the lines. However, for both kinds of lines, we could guarantee equal maintenance of the central line bundle. Finally, we suggest a multicenter, double-blind randomized control trial to gain even further insights into the issue because the observational study, in addition to the small sample size, might not be sufficient to detect a meaningful difference.

## Conclusions

In the current study, we conclude that femoral lines don't increase the risk of CRBSI when compared to internal jugular lines. Moreover, avoiding femoral vascular access will not reduce the burden of CRBSI at the population level if the central line bundle is properly maintained. When inferring causal relationships, inverse probability treatment weighting is a very useful technique. Further work should be focused on randomized, double-blind, multicenter studies to establish causality on this crucial question.

## Data Availability

The datasets used and analyzed during the current study are available from the corresponding author on reasonable request.

## Abbreviations

ANOVA: Analysis of variance
CDC: The Centers for Disease Control and Prevention
CLABSI: Central lines associated blood stream infection
CRBSI: Catheter-related bloodstream infection
CVC: Central venous catheters
DAGs: Directed acyclic graphs
DTP: Time to positivity
HICPAC: Healthcare Infection Control Practices Advisory Committee
IPTW: Inverse probability treatment weighting
IRB: Institutional Review Board
NHSN: National Healthcare Safety Network
PICC: Peripherally inserted central catheter
SMD: Standardized mean difference
TPN: Total parenteral nutrition
